# Development of an Orthotopic Human Pancreatic Cancer Xenograft Model Using Ultrasound Guided Injection of Cells

**DOI:** 10.1371/journal.pone.0020330

**Published:** 2011-05-27

**Authors:** Amanda Shanks Huynh, Dominique F. Abrahams, Monica S. Torres, Margaret K. Baldwin, Robert J. Gillies, David L. Morse

**Affiliations:** 1 Department of Functional and Molecular Imaging, H. Lee Moffitt Cancer Center and Research Institute, Tampa, Florida, United States of America; 2 Division of Comparative Medicine, University of South Florida, Tampa, Florida, United States of America; University of California, Los Angeles, and Cedars-Sinai Medical Center, United States of America

## Abstract

Mice have been employed as models of cancer for over a century, providing significant advances in our understanding of this multifaceted family of diseases. In particular, orthotopic tumor xenograft mouse models are emerging as the preference for cancer research due to increased clinical relevance over subcutaneous mouse models. In the current study, we developed orthotopic pancreatic cancer xenograft models in mice by a minimally invasive method, ultrasound guided injection (USGI) comparable to highly invasive surgical orthotopic injection (SOI) methods. This optimized method prevented injection complications such as recoil of cells through the injection canal or leakage of cells out of the pancreas into the peritoneal cavity. Tumor growth was monitored in vivo and quantified by ultrasound imaging weekly, tumors were also detected by in vivo fluorescence imaging using a tumor targeted molecular probe. The mean tumor volumes for the USGI and SOI models after 2 weeks of tumor growth were 205 mm^3^ and 178 mm^3^ respectively. By USGI of human pancreatic cancer cell lines, human orthotopic pancreatic cancer xenografts were established. Based on ultrasound imaging, the orthotopic human pancreatic cancer xenograft take rate was 100% for both human pancreatic cancer cell lines used, MiaPaCa-2 and Su86.86, with mean tumor volumes of 28 mm^3^and 30 mm^3^. We demonstrated that this USGI method is feasible, reproducible, facile, minimally invasive and improved compared to the highly-invasive SOI method for establishing orthotopic pancreatic tumor xenograft models suitable for molecular imaging.

## Introduction

Mice have been employed as models of cancer for over a century, providing significant advances in our understanding of this multifaceted family of diseases. There are currently four main areas of cancer research that use mouse models: basic biology and physiology, experimental therapeutics, prevention, and genetics susceptibility and risk. These models have proven to be useful in validation of gene function, characterization of novel cancer genes and tumor biomarkers, gaining insight into the molecular and cellular mechanisms underlying tumor initiation and multistage processes of tumorigenesis, and providing better clinical models in which to test novel therapeutic strategies [1]. In particular, tumor xenograft mouse models are commonly used in preclinical studies. Human tumor xenograft models are created by the injection of human tumor cells grown from culture into a mouse or by the transplantation of a human tumor mass into a mouse. The xenograft may be readily accepted by immunocompromised mice such as athymic nude mice or severely compromised immunodeficient (SCID) mice [2]. There are several key advantages of using human tumor xenografts: they feature the complexity of genetic and epigenetic abnormalities that exist in the human tumor population; can be used to aid in the development of individualized molecular therapeutic approaches; and can be implanted orthotopically to reproduce the organ environment in which the tumor grows, so that the effect of the tumor on its microenvironment can be modulated [3].

The two main types of human xenograft mouse models used for cancer research, heterotopic and orthotopic are defined by the location of the implanted xenograft. For heterotopic subcutaneous models, the xenograft is implanted between the dermis and underlying muscle and is typically located on the flank, on the back or the footpad of the mouse. For over 30 years, the subcutaneous xenograft model has been the most widely used preclinical mouse model for cancer research because it is rapid, inexpensive, easily reproducible, and has been considered sufficiently preclinical to test anti-cancer drugs. The subcutaneous model also has the advantages of providing visual confirmation that mice used in an experiment have tumors prior to therapy; and providing a means of assessing tumor response or growth over time, compared to intracavitary models where animal survival is the sole measure of response [4]. However, major disadvantages in the preclinical use of subcutaneous xenograft models have become evident. It has been consistently observed that drug regimens that are curative in mouse subcutaneous xenograft models often do not have a significant effect on human disease. The primary cause of this failure may be due to the observation that the subcutaneous microenvironment is not relevant to that of the organ site of primary or metastatic disease. Additionally, subcutaneous tumor models rarely form metastases. These observations suggest that heterotopic tumor models that do not represent appropriate sites for human tumors are not predictive when used to test responses to anti-cancer drugs [5].

To address the deficiencies of subcutaneous models, orthotopic tumor xenografts are increasingly being explored for increased clinical relevance. In this model, the tumor xenograft is either implanted or injected into the equivalent organ from which the cancer originated, or where metastases are found in patients. Advantages of orthotopic models include use of the relevant site for tumor-host interactions, the emergence of disease-relevant metastases, the ability to study site-specific dependence of therapy, organ-specific expression of genes and that clinical scenarios can be replicated, e.g. surgical removal of primary tumor, or adjuvant therapy of occult metastasis [5]. Major disadvantages are that orthotopic tumor xenograft generation is labor intensive, technically challenging, expensive, requiring longer healing and recovery time and that monitoring tumor volume requires relatively lower throughput imaging methods compared to the use of calipers [5]. Nonetheless, orthotopic tumor models are emerging as the preference for cancer research due to the increased clinical relevance.

There is a strong need to develop improved models for the pre-clinical investigation of pancreatic cancer. It is estimated that 43,140 people (21,370 men and 21,770 women) will be diagnosed with cancer of the pancreas in 2010, and that 36,800 men and women will die of this disease [6]. The prognosis is poor, with fewer than 5% survival five years after diagnosis, and complete remission is rare. No effective early detection methods have been developed and progress in development of treatment and therapy is stagnant. Gemcitabine has been the standard chemotherapy for more than a decade. However, the benefit of single-agent gemcitabine therapy in advanced and metastatic pancreatic cancer is small [7]. The use of orthotopic xenograft models for preclinical pancreatic cancer research may improve the development of therapies and diagnostic imaging modalities against this disease.

In this study, we investigated the feasibility of developing orthotopic human pancreatic cancer xenograft models using ultrasound guided injection (USGI) of tumor cells. Orthotopic tumor xenograft mouse models for pancreatic cancer have been well established for many years. However, these models require the highly invasive surgical orthotopic implantation (SOI) of tumor cells or chunks into the pancreas. This procedure can result in significant trauma, requiring post-operative recovery time, and can lead to adverse events such as infection, bleeding, tumor adhesion to other organs, and other effects from the surgical stress, e.g. wound healing.

Recent advancements have allowed for the development of image-guided methods for orthotopic injection of cells or tissue into internal organs, potentially eliminating the need for highly invasive surgical procedures. In particular, minimally invasive real-time ultrasound guided injection (USGI) of tumor cells can be performed to create orthotopic xenograft models. USGI of tumors cells has become an accepted method for developing orthotopic hepatocellular carcinoma models. For example, a multi-drug resistant model was successfully established in nude mice via orthotopic implantation of multi-drug resistant human HCC cells directed by ultrasonography [8]. We have successfully developed a novel orthotopic xenograft model for lymph node metastasis, where precise numbers of tumor cells were injected into the axillary lymph nodes using ultrasound image guidance [9].

A syngeneic orthotopic murine pancreatic cancer mouse model was developed by injection of murine pancreatic ductal adenocarcinoma cells, Pan02, into or near the pancreas of C57BL/6 mice using SonoCT ultrasound guidance [10]. The ultrasound guided method was concluded to be favorable compared to the subcutaneous model for the investigation of the influence of immunotherapy on tumor growth [10]. However, tumors resulting from this study appeared to be widely disseminated throughout the abdominal cavity and not isolated solely to the pancreas, and it was not clear whether this was a result of metastasis, or possibly cells being released into the surrounding area during the procedure.

Our current study is the first to report the orthotopic injection of human pancreatic cancer cells directly into the pancreas of immunocompromised mice by ultrasound-image guidance. We have fully characterized the tumor take rates relative to the surgical model and have confirmed by histology that the tumors were initially isolated to the pancreas followed by subsequent metastasis into the peritoneal cavity in later weeks. Additionally, we have demonstrated that a targeted molecular imaging agent can be specifically delivered through the vasculature to the resulting tumors in the pancreas.

## Materials and Methods

### Ethics Statement

All procedures were in compliance with the Guide for the Care and Use of laboratory Animal Resources (1996), National Research Council, and approved by the Institutional Animal Care and Use Committee, University of South Florida under the approved protocol R3715. Immunocompromised mice are housed in a clean facility with special conditions that include HEPA filtered ventilated cage systems, autoclaved bedding, autoclaved housing, autoclaved water, irradiated food and special cage changing procedures. Mice are handled under aseptic conditions including the wearing of gloves, gowns and shoe coverings.

### Cell Culture

MiaPaca-2 cells (ATCC CRL-1420), SU86.86 cells (ATCC CRL-1837), and the parental HCT116 cells (ATCC CCL-247) were purchased from ATCC (Manassas, VA). The HCT116/δOR+ colon cancer cells were genetically engineered from the parental HCT116 cell line to highly over-express the δ-opioid receptor [11]. Expression of the δOR on the surface of HCT116 and HCT116/δOR+ cells was characterized prior to injection using an *in vitro* time-resolved fluorescence binding assay [12]. HCT116/δOR+, and MiaPaCa-2 cells were cultured in DMEM/F12 media (Invitrogen, Carlsbad, CA) supplemented with 10% normal calf serum (Atlanta Biologicals, Lawrenceville, GA) and 1% penicillin/streptomycin solution (Sigma, St. Louis, MO). The SU86.86 cells were cultured in RPMI media (Invitrogen) supplemented with 10% FBS (Atlanta Biologicals). All cells were grown at 37°C and 5% CO2.

### Surgical Orthotopic Pancreatic Cancer Xenograft Mouse Model

For comparison to the USGI technique, 5 female nu/nu mice 6-8 weeks old (Harlan Sprague Dawley Inc., Indianapolis, IN) underwent an established surgical method for orthotopic injection of cells into the pancreas. For this procedure, mice were anesthetized under isoflurane gas; the abdominal skin and muscle were incised just off the midline and directly above the pancreas to allow visualization of the pancreatic lobes; the pancreas was gently retracted and positioned to allow for direct injection of a 20 µL bolus of 1×10^6^ HCT116/δOR+ cells/PBS using a 1 cc syringe with a 30 gauge needle; successful delivery of cells into the pancreas was observed under magnification using a dissection microscope; the pancreas was placed back within the abdominal cavity; and both the muscle and skin layers closed with surgical glue. Following recovery from surgery, mice were monitored and weighed daily.

## Ultrasound Imaging

The Visual Sonics Vevo 2100 Imaging Station was used for all ultrasound imaging. Image acquisitions were performed using the enhanced abdominal measurement package in the B-mode and 3-D mode settings. Physiological status (ECG, respiration, blood pressure, and body temperature) of the mice was closely monitored during each image acquisition session. Mice were imaged prior to tumor xenografting to establish baseline images and then imaged weekly for up to 4 weeks using ultrasound to monitor development of the orthotopic pancreatic cancer xenografts.

### Fluorescence Imaging

Mice bearing orthotopic pancreatic xenografts of the HCT116/δOR+ cells were administered 4.5 nmol/kg body weight of Dmt-Tic-Cy5 by tail vein injection. Dmt-Tic-Cy5 is a high affinity (3 nM Ki) peptidomimedic targeted probe conjugated to fluorescent dye (Cy5) that was used to determine the location of the HCT116/δOR+ cells by *in vivo* and *ex vivo* fluorescence imaging [12]. Following injection, mice were kept in a special dark chamber and protected from light exposure as much as possible to prevent photo bleaching of the dye. Alfalfa-free food and special cage bedding were used to minimize autofluorescence.

*In vivo* and *ex vivo* fluorescence images were acquired using the Caliper Life Sciences Xenogen IVIS 200 Series Imaging System. The 615–665 nm excitation and 695–770 nm emission filters were used. Acquisition times ranged from 0 s to 10 s to keep intensity counts within the range of 15,000 to 60,000 to prevent saturation. Prior to data analysis, instrument background subtractions were performed. Living Image 3.2 Software was used to draw regions of interest (ROIs) over the tumors to determine the mean fluorescence signal (efficiency units). Efficiency units are calculated by normalizing fluorescence emission images for variations in the incident excitation light distribution on the stage. Autofluorescence background was subtracted by determining the mean tumor fluorescence signal prior to injection.

### Histology of Pancreatic Xenografts

The pancreata of the mice were harvested, visually inspected, fixed in 10% formalin buffer, processed, embedded, tri-sectioned, H&E stained, and analyzed by a pathologist for the presence of tumors.

### Statistics

Data are represented as mean ± s.d. and Student’s t-test was used to determine significance.

## Results

### Development of the Orthotopic Human Pancreatic Cancer Xenograft Mouse Model Using USGI

**Figure 1:**
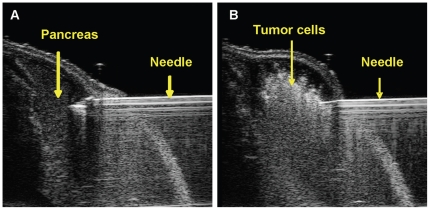
Representative ultrasound images of USGI of tumor cells into the pancreas. A) Shows the 30 gauge needle in mouse pancreas pre-injection. B) Shows the injection of a 20 µL bolus of tumor cells into the mouse pancreas.

The orthotopic human pancreatic cancer mouse model was developed using 6–8 week old female athymic nude mice. Mice were anesthetized using 3% isoflurane gas via induction chamber and then secured in dorsal recumbency on the ultrasound platform with a nose cone for maintenance of anesthesia at 1.5 to 2%. The ultrasound platform is positioned to have the pancreas side of the body towards the mechanical needle holder. Ultrasound gel was applied to the abdomen and the pancreas located by mechanically adjusting the position of the ultrasound transducer, with the spleen as a reference. A prescan image of the mouse abdominal region was performed using the 3D mode imaging acquisition feature prior to xenografting to establish a baseline for comparative analysis. A 0.5 mL syringe with a 30 g needle was placed in the mechanical syringe holder and lined up parallel to the probe and perpendicular to the body. The syringe needle was then properly aligned and advanced into the pancreas using the needle guide overlay feature that allows for the visualization of the needle alignment and injection target on the US monitor. A 20 µL bolus of 1×10^6^ tumor cells suspended in PBS was injected directly into the pancreas using the automated image-guided precision micro-injection feature. An optimized injection technique was used, in which the injection volume was decreased from an initial volume of 50 µL to 20 µL. Instead of immediately retracting the needle, a 5 second pause after the cell injection with a slow, deliberate withdrawl of the needle allowed for complete delivery of cells into the pancreas. The optimized technique prevented injection complications such as recoil of cells through the injection canal or leakage of cells out of the pancreas into the peritoneal cavity. Figure 1 shows the real-time ultrasound acquired images of the USGI of 1×10^6^ HCT116/δOR+ cells in a 20 µL bolus into the mouse pancreas. Once an injection was completed, the anesthesia was discontinued and the mouse was returned to the original housing and observed until capable of purposeful movement. The physiological status (heart rate, body temperature, ECG and respiration rate) of the mice was tracked during the entire process using the Vevo’s Advanced Physiological Monitoring Unit. Following USGI xenografting, mice were monitored and weighed daily to identify any signs of stress or trauma due to the procedure and or tumor burden, and no significant weight loss or other trauma was observed.

The HCT116/δOR+ model was chosen because we have developed a highly specific molecular imaging probe (Dmt-Tic-Cy5) for detection of cells expressing the human δ-opioid receptor and have previously used this probe for the *in vivo* and *ex vivo* specific detection of HCT116/δOR+ cells by fluorescence imaging [12]. Although not orthotopic, use of this line is also relevant because colorectal cancer is known to form metastases in the pancreas [13].

**Figure 2:**
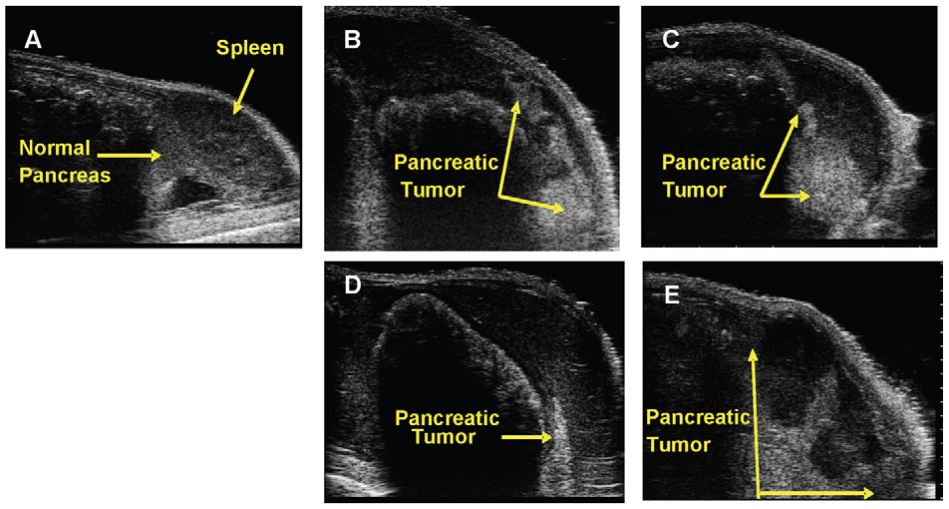
Representative ultrasound images of orthotopic human pancreatic cancer xenografts in the mouse pancreas over time. A) Normal mouse pancreas, B) 1 week post-USGI, C) 2 weeks post-USGI, D) 1 week post-SOI, and E) 2 weeks post-SOI.

Initially mice bearing orthotopic xenografts of HCT116/δOR+ via SOI and USGI were imaged weekly by ultrasound for up to 4 weeks to monitor tumor growth. The 3D-mode imaging acquisition feature was used, in which a 3D motor translates the Microscan array transducer across the abdomen to obtain multiple 2D slices that are assembled and rendered by the Vevo software into a 3D data set of anatomical structures of the pancreas and other abdominal organs. Figure 2 shows ultrasound images of mouse pancreata bearing tumor xenografts acquired at time 0 (prior to injection of cells), 1 week and 2 weeks post- USGI and SOI of cells. After 3 to 4 weeks, mice had extremely large tumors with swollen distended abdomens, and at these later time-points, some animals had metastases in various regions of the abdominal cavity, e.g. the GI tract, liver, and lungs.

**Figure 3:**
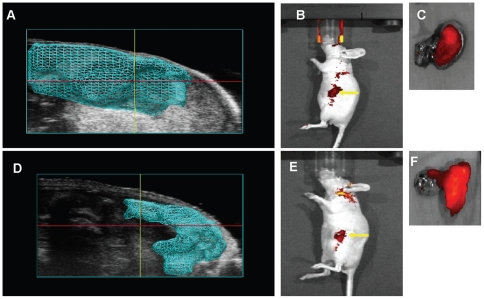
Comparison of representative images of SOI and USGI orthotopic pancreatic cancer xenografts models 2 weeks post-injection of HCT116/δOR+ cells. The blue shaded area represents the tumor generated by performing 3D tumor volume measurements. A) ultrasound image of USGI model, B) *in vivo* fluorescence image of USGI model, C) *ex vivo* fluorescence image of the mouse pancreas from the USGI model, D) ultrasound image of SOI model, E) *in vivo* fluorescence image of SOI model, and F) *ex vivo* fluorescence image of the mouse pancreas from the SOI model.

**Figure 4:**
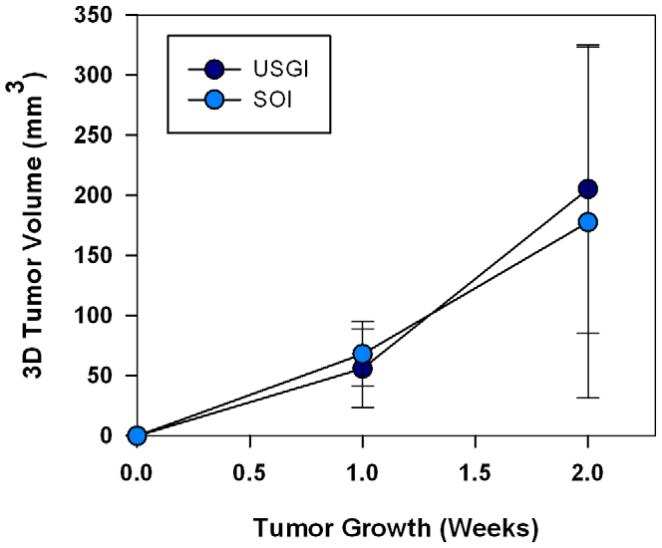
Mean 3D tumor volume measurements (n = 5) from the USGI and SOI models of orthotopic pancreatic cancer xenografts growth over time.

**Figure 5:**
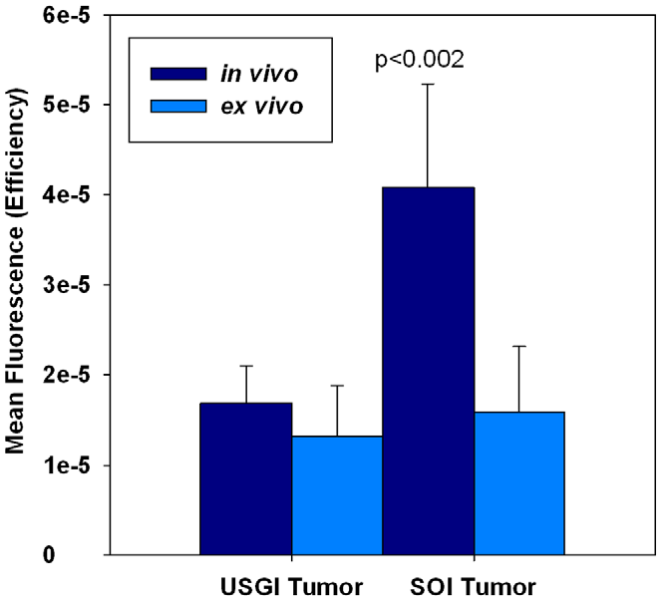
Mean normalized fluorescence (n = 5) from *in vivo* and *ex vivo* images for HCT116/δOR+ USGI and SOI orthotopic pancreatic cancer xenografts 24 h post-injection of fluorescent probe.

To demonstrate the feasibility of developing an orthotopic pancreatic model by USGI that is comparable to SOI methods, 5 mice each underwent USGI or SOI of 1×10^6^ HCT116/δOR+ cells into the pancreata. Tumor growth was monitored and quantified by ultrasound imaging weekly. After 2 weeks, large pancreatic xenograft tumors were observed in all 10 mice by ultrasound imaging (Figure 3). 3D volume measurements were quantified by using the 3D-Mode Volume tool, in which volumes are created by drawing contours around the tumor in every 10 slices the series of images to create a 3D tumor volume and image. Figure 3 shows representative 3D ultrasound images of the USGI (3A) and SOI (3D) mouse xenograft models 2 weeks after injection of tumor cells. Mean tumor volumes (n = 5) for the USGI and SOI models were plotted over time in Figure 4 and are recorded in Table 1. Mice were then administered 4.5 nmol/kg Dmt-Tic-Cy5 probe via tail vein injection. *In vivo* fluorescence images were acquired 24 h later, which provided maximum contrast. The *in vivo* fluorescence images in Figure 3 showed uptake of the fluorescent probe in the area of the pancreas for both mouse models, indicating the presence of HCT116/δOR+ tumor cells. The *in vivo* mean fluorescence signal (n = 5) was 2-fold higher for SOI compared to the USGI model, p<0.002 (Figure 5). Based on *in vivo* ultrasound and fluorescence imaging, the take rate was 100% for both models while using HCT116/δOR+ tumor cells.

**Table 1:** Mean 3D volume measurements of orthotopic pancreatic cancer xenografts.

Xenograft Model	Week 1		Week 2	
	Mean (mm^3^)	S.D.	Mean (mm^3^)	S.D.
USGI	56.0	32.7	205.0	120.0
SOI	68.1	27.0	177.5	145.8
SU86.86	12.9	6.6	28.9	12.1
MiaPaCa-2	11.6	6.5	28.2	5.3

**Figure 6:**
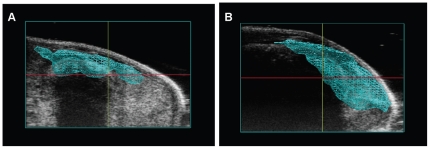
Representative ultrasound acquired images of orthotopic human pancreatic cancer xenografts 2 weeks after USGI of: A) MiaPaCa-2 cells B) SU86.86 cells. The blue shaded area represents the tumor generated by performing 3D tumor volume measurements.

**Figure 7:**
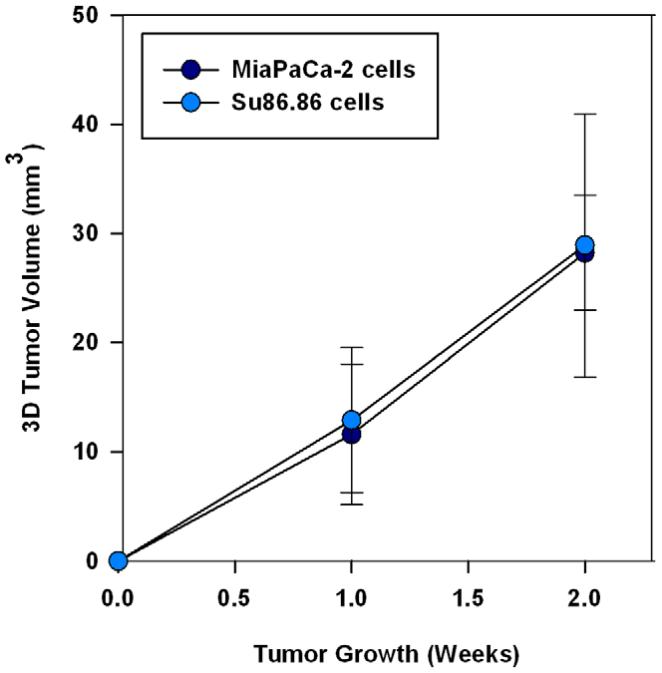
Mean 3D tumor volume measurements (n = 5) from the orthotopic human pancreatic cancer xenografts using pancreatic cancer cell lines, MiaPaca-2 and SU86.86, growth over time.

To determine if USGI can be used with human pancreatic cancer cell lines, 5 mice underwent USGI of 2×10^6^ MiaPaCa-2 cells in a 20 µL bolus and another 5 mice underwent USGI of 2×10^6^ SU86.86 cells. After 2 weeks, all mice had orthotopic pancreatic tumor xenografts observed by ultrasound imaging and visual inspection (Figure 6). Mean tumor volumes (n = 5) were plotted over time for the MiaPaCa-2 and Su86.86 human pancreatic tumors (Figure 7) and recorded in Table 1. Based on *in vivo* ultrasound imaging, the orthotopic xenograft take rate was 100% while using both human pancreatic cancer cell lines, MiaPaCa-2 and Su86.86.

### Validation by Ex vivo Fluorescence Imaging and Histology

**Figure 8:**
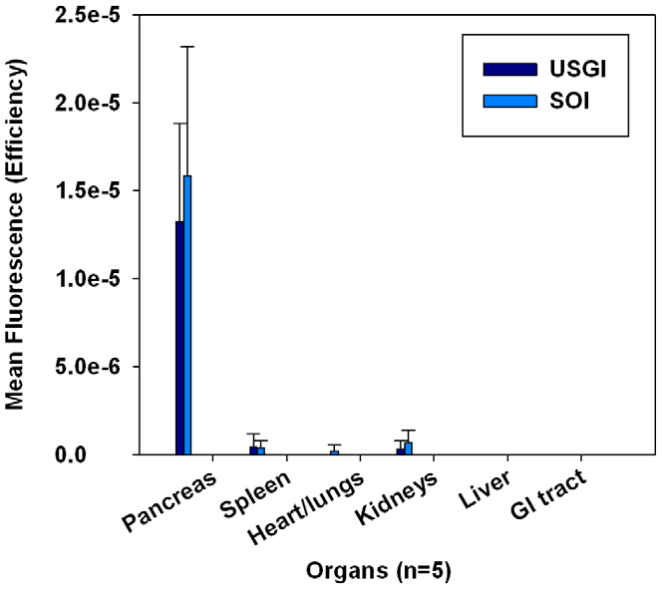
Mean *ex vivo* fluorescence signal from mouse organs (pancreas, spleen, heart, lungs, liver, kidneys, and GI tract) obtained 24 h post-administration of tumor targeted fluorescent-probe and 2 weeks post SOI or USGI, n = 5.

Immediately after *in vivo* fluorescence imaging, the HCT116/δOR+ tumor xeongraft bearing mice were humanely euthanized, visually examined for the presence of tumors elsewhere in the body, and major organs (heart, lung, kidneys, liver, spleen, pancreas, GI tract) removed, and *ex vivo* fluorescence images acquired 24 h post-administration of the targeted fluorescent probe. The *ex vivo* mean fluorescence (n = 5) acquired from pancreata of both models was relatively the same (Figure 5). As determined by *ex vivo* imaging, probe specific fluorescence was present in 100% of the mouse pancreata (n = 5) for both models, and a low level of fluorescence was observed in the kidneys (Figure 8).

**Figure 9:**
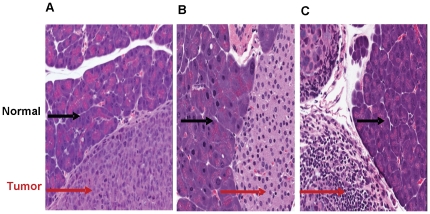
Representative H&E staining of mouse pancreata containing xenografts of the following cell lines: A) HCT116/δOR+, B) MiaPaca-2 and C) SU86.86. The 20X magnification view shows normal pancreas (indicated by black arrows) adjacent to tumor cells (indicated by red arrows).

By histology, tumors were observed in 100% of pancreata that underwent USGI or SOI using HCT116/δOR+ cells. Tumors were observed in only 4 out of 5 (80%) of pancreata injected with MiaPaCa-2 or SU86.86 cells, even though the take rate was 100% based on the US imaging. Histology from these pancreata was only prepared from 3 sections that were 50 µm apart, hence, it is probable that tumors were missed during sectioning. Figure 9 shows representative H&E staining of mouse pancreata containing tumor xenografts of all three cell lines. For HCT116/δOR+ cell xenografts, the mean percentage (n = 5) of malignant tissue relative to unaffected tissue found in a single section of the pancreas was 76±15 % for the SOI method and 62±13 % for USGI (Table 2). Each xenograft was histologically graded for type of malignancy and mean percentage scored (n = 5) for SOI and USGI respectively: 84%±9 and 85%±9 cellular, 12%±5 and 11%±6 stromal, and 4%±4 and 4%±3 necrotic (Table 2).

**Table 2:** Pathological grading of malignancy in 10 orthotopic pancreatic cancer xenografts established with HCT116/δOR+ cells.

Mouse	Tumor Cell	Normal	Malignancy	Malignancy Type (%)		
	Injection Method	(%)	(%)	Cellular	Stroma	Necrosis
1	SOI	20	80	90	8	2
2	SOI	15	85	80	15	5
3	SOI	20	80	90	7	3
4	SOI	15	85	70	20	10
5	SOI	50	50	90	8	2
6	USGI	40	60	95	5	0
7	USGI	30	70	85	10	5
8	USGI	30	70	90	8	2
9	USGI	60	40	70	20	10
10	USGI	30	70	85	10	5

## Discussion

To the best of our knowledge, this is the first account of orthotopic xenografting of human pancreatic cancer cells by USGI into the mouse pancreas. In this study, we demonstrated that this USGI method is feasible, reproducible, facile, minimally invasive and improved compared to the highly-invasive SOI method for establishing orthotopic pancreatic models suitable for molecular imaging applications. USGI is an improvement over the SOI method for imaging because the invasive portion of the procedure takes only 30 seconds versus 5 minutes and with a shorter recovery and healing time. The surgical wound was still present at the time of fluorescence imaging 2 weeks later with the SOI method, while the USGI needle prick wound was no longer visible after 1 day. The presence of the surgical wound altered the autofluorescence properties of the skin and can thus decrease the quality fluorescence imaging studies.

Ultrasound imaging was used to track the injection of tumor cells *in vivo*. The real-time visualization of the cell injections allowed for optimization of the injection method. In initial experiments, we observed cells spilling out of out the pancreas into the peritoneal cavity when a 50 µL bolus of tumor cells was injected directly into the pancreas by both the USGI and SOI method. This volume was chosen initially because it was the volume used in the syngeneic orthotopic pancreatic model developed by Schneider et al, in which they observed abdominal metastases in most cases [10]. We also observed abdominal metastases in mice receiving a 50 µL bolus and suspect that these metastases may be due to cell spillage. We also observed recoil of cells through the injection canal when the needle was removed from the pancreas too quickly, which resulted in 3 mice developing a small subcutaneous tumor at the injection site. Therefore, we optimized the injection volume to 20 µL and the injection method to prevent spillage and recoil of cells out of the pancreas.

Initial experiments determined that orthotopic pancreatic xenografts of HCT116/δOR+ cells exhibit peritoneal dissemination after 3 to 4 weeks, which has been reported by others as well [14], [15], [16]. In this time frame, we observed metastases to the peritoneum, liver, and lungs. Metastases were not detected at 2 weeks. Others have observed significant metastasis to the peritoneum, liver, lungs, and lymph nodes following surgical orthotopic injection of tumor cells into the pancreas [17]. This suggests that pancreatic cancer xenografts using HCT116/δOR+ cells are similar to pancreatic cancer cell xenografts. Since we were investigating the feasibility of USGI by determining tumor xenograft take rate and not metastasis, we chose to carry out the rest of the study using mice with 2 week-old orthotopic pancreatic cancer xenografts.

Ultrasound and fluorescence imaging were used to identify the location of tumors and to monitor the progress of tumor growth *in vivo*. Although HCT116/δOR+ cells are human colorectal cancer cells, they are relevant for pancreatic xenograft models because they are known to form occult metastases in the pancreas [13]. Furthermore, they could be tracked by a molecular imaging probe we had previously developed, the peptidomimetic fluorescent probe (Dmt-Tic-Cy5) that specifically targets the δ-opioid receptor expressed on the surface of this tumor cell line with sub nM affinity [12].

The success in generation of orthotopic pancreatic xenograft tumors using USGI was comparable to our SOI methods using HCT116/δOR+ cells. Based on ultrasound imaging, *in vivo* and *ex vivo* fluorescence imaging, and histological analysis, the xenograft take rate was 100% for both USGI and SOI using HCT116/δOR+ cells. For both models, there was a strong correlation in observed tumor formation using both ultrasound and fluorescence imaging. Snyder et al., have also reported that fluorescence and ultrasound imaging modalities are complementary approaches for monitoring tumor progression and treatment response in preclinical studies using orthotopic mouse models of human pancreatic cancer [18]. The tumor xenograft volumes for SOI and USGI were comparable at both 1 and 2 weeks post-implantation. However, the quantified mean fluorescence signal (n = 5) acquired from *in vivo* imaging of the SOI model was 2 fold higher in enhancement compared to the USGI model while there was no significant difference in the quantified *ex vivo* signals. The increased *in vivo* fluorescence signal generated from the SOI models relative to *ex vivo* signal may be due to surgical complications, such as trauma of the tissue in this region causing wound healing. The wound at the surgical injection site was still present at the time of fluorescence imaging, which may have increased fluorescent properties compared to unaffected tissue. Regardless, *in vivo* fluorescence imaging of the USGI model was superior to SOI, as the quantified *in vivo* signal was equivalent to the *ex vivo* signal obtained from the pancreas. At the 24 hr time-point post injection of targeted fluorescent probe, a low level of fluorescence was observed in the kidneys, spleen, heart and lungs. However the SOI model had significantly greater signal, p<0.002, in these tissues compared to USGI. This is possibly due to increased metastasis from xenografts generated by SOI relative to USGI at the same time-point post injection of cells (2 weeks). Based on the average amount of malignancy relative to normal pancreas tissue as determined by the pathologist (Table 1), the 2 methods yielded nearly equal types of malignancy; with an average of 85% cellular, 11% stromal and 4% necrotic components. Hence, we have demonstrated that orthotopic pancreatic tumor xenografting by USGI is feasible and comparable to results obtained by SOI.

Since the initial phases of the study used colorectal cancer cells, two human pancreatic cell lines, MiaPaCa-2 and Su86.86, were chosen to demonstrate the feasibility of developing orthotopic human pancreatic tumor xenografts by USGI. These cell lines have previously been used for establishing surgical orthotopic models of pancreatic cancer [19], [20], [21]. Based on the ultrasound images acquired after 2 weeks, the tumor xenograft take rate was 100% and the measured volumes were similar. Our take rate is at the high-end of rates reported in the literature, e.g. Ding et al. reported that the rate of tumor establishment in the pancreas can be anywhere from 50 to 100% using orthotopic transplantation [22]. However, it is possible that in the negative pancreata tumors were present in other sections not histologically examined.

Confirmation of tumor xenografts by fluorescence imaging could not be performed because there are currently no fluorescent probes available that specifically target these cells. However, Hausner and colleagues have demonstrated targeted *in vivo* imaging of subcutaneous pancreatic tumor xenografts by positron emission tomography (PET) using the α_v_β_6_-specific [^18^F]FBA-PEG_28_-A20FMDV2 peptide agent [23]. The specificity of this PET agent could be explored in an orthotopic pancreatic cancer model by USGI of α_v_β_6_-expressing BxPC-3 cells and the non-expressing MiaPaca-2 cells [23].

In conclusion, the USGI orthotopic human pancreatic cancer xenograft model is both plausible and comparable to the traditional but more invasive SOI model in developing orthotopic models of pancreatic cancer. We have developed and optimized a minimally invasive, rapid and reproducible method for the generation of orthotopic human pancreatic cancer xenograft mouse models by ultrasound-guided injection (USGI) of tumor cells directly into the pancreas for use in molecular imaging of cancer research.
